# The Environmental Enteric Dysfunction Biopsy Initiative (EEDBI) Consortium: mucosal investigations of environmental enteric dysfunction^☆^

**DOI:** 10.1016/j.ajcnut.2024.02.003

**Published:** 2024-09-17

**Authors:** Donna M Denno, Sheraz Ahmed, Tahmeed Ahmed, S Asad Ali, Beatrice Amadi, Paul Kelly, Sarah Lawrence, Mustafa Mahfuz, Chelsea Marie, Sean R Moore, James P Nataro, William A Petri, Peter B Sullivan, Phillip I Tarr, EEDBI Consortium, Kumail Ahmed, Md Ashraful Alam, Barrett H Barnes, SM Khodeza Nahar Begum, Stephen M Borowitz, Kanta Chandwe, Miyoba Chipunza, Subhasish Das, Lee A Denson, Jeffrey R Donowitz, Shah Mohammad Fahim, Md Amran Gazi, Carol A Gilchrist, Rashidul Haque, Md Mehedi Hasan, Md Shabab Hossain, Aneeta Hotwani, Junaid Iqbal, Najeeha Talat Iqbal, Sadaf Jakhro, Furqan Kabir, Barbara J Mann, Ramendra Nath Mazumder, Waheeda Memon, Jeremy P Middleton, Uma Nayak, Sandra Oliphant, Abdul Khalique Qureshi, Masudur Rahman, Najeeb Rahman, Girija Ramakrishnan, Kamran Sadiq, Shafiqul Alam Sarker, Fayaz Umrani

**Affiliations:** 12 Department of Paediatrics and Child Health, Aga Khan University, Karachi, Pakistan; 13 International Centre for Diarrhoeal Disease Research, Bangladesh, Dhaka, Bangladesh; 14 Department of Pediatrics, University of Virginia Children's Hospital, University of Virginia, Charlottesville, VA, United States; 15 Department of Pathology, Bangladesh Specialized Hospital, Dhaka, Bangladesh; 16 Tropical Gastroenterology & Nutrition Group, University of Zambia School of Medicine, Lusaka, Zambia; 17 Division of Pediatric Gastroenterology, Hepatology, and Nutrition, Cincinnati Children's Hospital Medical Center, Cincinnati, OH, United States; 18 Division of Pediatric Infectious Diseases, Children's Hospital of Richmond at Virginia Commonwealth University, Richmond, VA, United States; 19 Department of Medicine, University of Virginia, Charlottesville, VA, United States; 20 Infectious Diseases Division, International Centre for Diarrhoeal Disease Research, Bangladesh, Dhaka, Bangladesh; 21 Department of Biological and Biomedical Sciences, Aga Khan University, Karachi, Pakistan; 22 Department of Public Health Sciences, University of Virginia, Charlottesville, VA, United States; 23 Department of Gastroenterology, Sheikh Russel National Gastroliver Institute and Hospital, Dhaka, Bangladesh; 1 Department of Pediatrics, University of Washington, Seattle, WA, United States; 2 Department of Paediatrics and Child Health, Aga Kahn University, Karachi, Pakistan; 3 Nutrition Research Division, International Centre for Diarrhoeal Disease Research, Bangladesh, Dhaka, Bangladesh; 4 Tropical Gastroenterology & Nutrition Group, University of Zambia, Lusaka, Zambia; 5 Blizard Institute, Barts & The London School of Medicine, Queen Mary University of London, London, United Kingdom; 6 Department of Medicine, University of Virginia, Charlottesville, VA, United States; 7 Department of Pediatrics, University of Virginia, Charlottesville, VA, United States; 8 Department of Medicine, University of Virginia, Charlottesville, VA, United States; 9 Department of Paediatrics, Children's Hospital, University of Oxford, Oxford, United Kingdom; 10 Department of Pediatrics, Washington University, St. Louis, MO, United States

**Keywords:** children, Bangladesh, Pakistan, Zambia, environmental enteric dysfunction, undernutrition

## Abstract

Environmental enteric dysfunction (EED) is an asymptomatic acquired disorder characterized by upper small bowel inflammation, villus blunting, and gut permeability. It is a major contributor to poor growth in childhood as well as other highly consequential outcomes such as delayed neuorcognitive development. After decades of intermittent interest in this entity, we are now seeing a resurgence in the field of EED. However, recent studies have been hampered by a lack of investigation of the target tissue—the upper small bowel. In 2016, the EEDBI (Environmental Enteric Dysfunction Biopsy Initiative) Consortium was established as a common scientific platform across 3 independent EED biopsy cohort studies in Bangladesh, Pakistan, and Zambia. Two centers in the United States recruited comparison groups of children undergoing endoscopy for clinical indications. The EEDBI Consortium goal was to augment the contributions of the individual centers and answer high-level questions amenable to analysis and interpretation across the studies. Here, we describe the Consortium and its cohorts and recruitment procedures across studies. We also offer details applicable to all papers in this supplement, which describe EED mucosal histology, morphometry, immunohistochemistry, and transcriptomics as well as histology relationship to pathogens and biomarkers.

## Background

Environmental enteric dysfunction (EED) is an asymptomatic enteropathy characterized by upper small bowel inflammation, villus flattening, malabsorption, and permeability identified by small bowel biopsies was described in the mid-20th century in Asian and African adults [[1], [2], [3], [4], [5]] and United States Peace Corps volunteers and military staff residing in these regions [6,7]. Remarkably, small bowel lesions in young adults resolved when they were repatriated to North America [8,9].

The recognition that childhood enteropathy in low- and middle-income countries (LMICs) is common and associated with suboptimal growth came about more recently. Campbell et al. [10] demonstrated that growth faltering in Gambian infants was accompanied with increased intestinal permeability, endotoxemia, and systemic inflammation, providing possible mechanistic underpinnings to the observations of Lunn et al. [11], who linked decreased gut barrier function to suboptimal growth. Goto et al. [12,13] recapitulated these findings in stunted children in Bangladesh and Nepal. Subsequently, Campbell et al. [14] reported blunted villi, hyperplastic crypts, and CD25+ T-cell infiltration suggesting a Th1 response in underweight and variably stunted Gambian children.

The condition was initially termed tropical enteropathy, because of its geographic distribution. However, in recent years, the term “environmental enteropathy” has been proposed to emphasize that the precipitant is environmental rather than related to geography per se [15]. Brunser et al. [16], for example, described the condition to be prevalent in nontropical Chile. Most recently, the term “environmental enteric dysfunction” (EED) has been used to encompass the disorder’s functional deficits [17].

EED has major consequences for populations in LMICs. First, poor linear growth in children, a highly prevalent form of undernutrition, is frequently accompanied by EED. Globally, 22% of children under the age of 5 y are stunted, defined as having height-for age *z*-score (HAZ) or length-for-age *z*-score (LAZ) < −2.0 [18]. The prevalence of stunting is the highest in South Asia and sub-Saharan Africa (32%) [18]. Stunted children are at risk of increased mortality risk, in 1 study >2-fold during acute illness episodes compared with children with an HAZ of ≥−1, and 5.5-fold among children with severe stunting (HAZ < −3) [19]. Even children with HAZ −2 to <−1 exhibited a 1.6-fold increased mortality risk. EED is also associated with delayed development. For example, systemic inflammation, potentially of gut origin, was identified as a determinant of poor neurocognitive development in a Bangladesh cohort, the majority of whom were stunted by age 2 y [20]. Stunting is also associated with long-term outcomes in adulthood, including chronic noncommunicable diseases and lost human potential and economic productivity [[21], [22], [23]].

The cause of EED is unknown, but colonization with specific enteric pathogens, exposures to fecally contaminated environments, and unfavorable gut microbial populations are widely considered to be its drivers. However, well-designed household hygiene and sanitization trials have demonstrated limited or no effect on enteropathy (as evidenced by biomarker analysis) or linear growth [[24], [25], [26]]. Similarly, nutritional interventions have been found to have quite modest positive effects on linear growth [26,27]. For example, a meta-analysis found that complementary feeding interventions increased mean LAZs among under-2s by 0.11 [28]. The mechanisms through which EED contributes to poor growth is thought to include malabsorption of nutrients and chronic systemic inflammation associated with gut inflammation and/or translocation of microbial products into the circulation.

## Rationale for the Environmental Enteric Dysfunction Biopsy Initiative Consortium

Although many early EED publications included assessments of intestinal biopsies, more recent work has lacked tissue-based data. This gap in knowledge is understandable; small bowel biopsy acquisition relied on a nonvisualized tethered capsule to obtain suction biopsies through most of the last century [[29], [30], [31]]. Pediatric fiberoptic endoscopic technology (with the ability to obtain forceps biopsies through the instrument under direct visualization) only became widely available in high-income countries in the 1980s. In view of the strong association between enteropathy and child growth and development in LMICs, small bowel tissue assessments are critical if we are to understand this association, and current technology now enables more sophisticated tissue analysis. First, tissue transcriptomics and systematic histopathology [including quantitative morphometry and immunohistochemistry (IHC)] can identify underlying pathophysiology and systematically characterize the lesion. Trialing treatments that lack a mechanistic basis, even if the treatment seems benign, can be an inefficient use of limited research resources. Second, in lieu of tissue-based assessments, the field must rely either on biomarkers of intestinal processes or EED outcomes of interest, particularly growth, as surrogate measures of EED. However, a biomarker specific to tissue injury in EED (akin to antibodies to tissue transglutaminase for celiac disease) has yet to be identified, and associations between biomarkers and tissue assessments have been rare. Third, though optimal growth is the ultimate goal of attempts to improve or prevent EED, growth as a surrogate outcome is problematic. Many factors in populations in which EED is prevalent, unrelated to gut injury, also contribute to poor growth, including food insecurity, micronutrient deficiencies, suboptimal breastfeeding, nonresponsive feeding practices, anorexia associated with infections, and increased metabolism from acute illness or injury. It is impossible to identify children who have EED in isolation of these other growth-threatening conditions or to adjust for each potential confounder. Hence, using growth as a proxy for EED risks type 2 error, that is, false determination of lack of association or efficacy if children were misclassified as having EED rather than another cause of poor growth.

Pinch forceps biopsies of upper small bowel tissue by esophagogastroduodenoscopy are frequently obtained in high-income settings to assess many pediatric disorders, and are generally considered safe. Chief risks relate to anesthesia, which can be greatly reduced by expert pediatric anesthesiology care and assiduous monitoring. A multicenter retrospective series reported endoscopic complications in 2.3% of 10,236 upper gastrointestinal endoscopies, mostly related to transient hypoxemia; none were serious. Bleeding was very rare, and not hemodynamically significant. The authors noted, however, that their survey might not have captured intramural duodenal hematomas, which remain rare events in children without obvious hemorrhagic diatheses [31], and which typically present several days after procedure. Notably, two of the enrolling centers in the current Consortium of studies experienced no complications in predecessor smaller scale biopsy studies nor were any complications reported among a comparison group of United States children undergoing endoscopy and biopsy for clinical purposes (Table 1). These studies resulted in the first systematic scoring system for EED, consisting of 11 histology parameters semiquantitatively scored and chosen to broadly and agnostically assess small bowel histopathology in the disorder [32].

**TABLE 1:** Phase 1 biopsy studies

Center	No. of children enrolled	Age at biopsy	Inclusion criteria
Agha Khan University (AKU), Karachi, Pakistan	10	<18 mo	Nonedematous wasting not requiring hospitalization, recruited from a community-based birth cohort nonresponsive to nutritional rehabilitation
University Teaching Hospital (UTH), Lusaka, Zambia	16	6–36 mo	Children hospitalized for the management of severe wasting and persistent diarrhea
Washington University in St. Louis	13	<18 y	Celiac disease based on serologic markers and histology
	6	<18 y	No serologic or histologic evidence of celiac disease, and diagnostic biopsy was considered to be normal

Because of the paucity of knowledge regarding EED pathophysiology and therapeutic targets, and based on data endorsing the high prevalence of this disorder and its adverse effects on children and populations, coupled with the promising data from the “Phase 1 studies” (Table 1), in 2015, the Bill and Melinda Gates Foundation (BMGF) issued a call for proposals for intestinal tissue-based studies of EED.

Three studies were funded as separately awarded grants (Table 2), so study designs, enrollment criteria and procedures, and intestinal and nonintestinal assessments varied; specifics by center are described below. However, the studies shared an overarching goal: advancing understanding of the pathophysiology of the disorder to inform biomarker development and therapeutic targets. In recognition of the common aims, in 2016 the BMGF formed a common scientific platform, the Environmental Enteric Dysfunction Biopsy Initiative (EEDBI) Consortium, to synergize the scientific endeavors across the studies. Although full harmonization of protocols was not possible, the Consortium sought common data from each center to the extent possible, and facilitated crosscenter data analyses and interpretation. There was divergence in inclusion criteria, biopsy handling after collection, RNA sequencing laboratories, time points relative to biopsy for the collection of less invasively obtained specimens (stool, urine, blood), enteric pathogen detection methods, and the selection of biomarkers and the ELISA kits for their measurement. However, there was a high degree of conformity in overall aims, ethical approach, and exclusion criteria. Histopathologic scoring, IHC methods and quantification, morphometric measurements, and statistical methods were completely harmonized. Despite some methodologic differences, we believe there was sufficient congruence to suggest a common clinical and pathophysiologic entity.

**TABLE 2:** Environmental Enteric Dysfunction Biopsy Initiative Consortium studies

Center	Study name	United States Center enrolling comparison groups
Agha Khan University (AKU), Pakistan	Study of Environmental Enteropathy and Malnutrition (SEEM)	Cincinnati Children’s Hospital Medical Center (CCHMC)
International Centre for Diarrhoeal Disease Research, Bangladesh (icddr,b), Bangladesh	Bangladesh Environmental Enteric Dysfunction (BEED)	University of Virginia (UVa)
University Teaching Hospital (UTH), Zambia	Biomarkers of Environmental Enteropathy in Children (BEECH)	—

**FIGURE 1:**
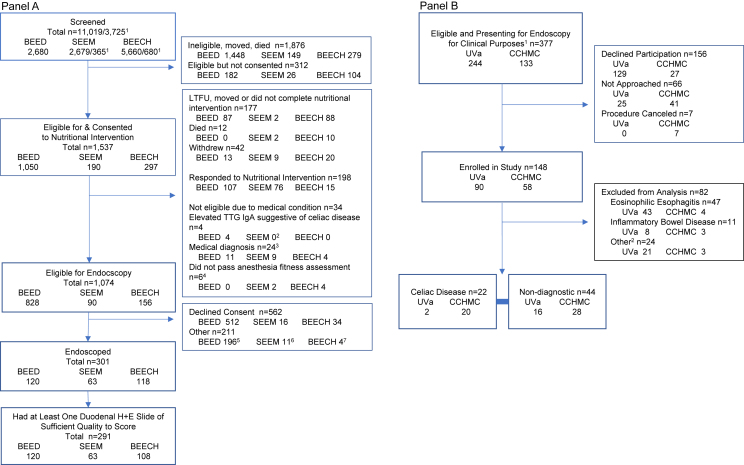
(A) BEED, SEEM, and BEECH study flow. ^1^SEEM: 2679 were screened as a birth cohort in the community and 365 were eligible at 3–6 mo for nutritional counseling and follow-up. BEECH: 5660 were screened in the community and based on WAZ < −1, 1792 were eligible for definitive health center-based screening. In total, 680 attended the full screening. ^2^Four SEEM participants had mildly elevated (≤2-fold of the upper limit of normal for the assay) serum TTG IgA concentrations on initial screen. On retesting ∼2 y later while consuming a local traditional diet, which typically contains gluten, these values had returned to normal. ^3^Cardiac condition (BEECH *n =* 2), renal condition (SEEM *n =* 1), seizure or other neurologic condition (SEEM *n =* 6), tuberculosis (BEECH *n =* 2, BEED *n =* 11, SEEM *n =* 2). ^4^Abnormal preanesthesia laboratory results [anemia (BEECH *n =* 1), leukocytosis (BEECH *n =* 1), elevated PT INR (BEECH *n =* 1), thrombocytopenia (SEEM *n =* 1), anesthesia-associated desaturation (BEECH *n =* 1), recurrent illnesses (SEEM *n =* 1)]. ^5^Not approached for endoscopy because ethical approval was limited to 120 endoscopies. ^6^>24 mo (*n =* 9), died (*n =* 1), moved (*n =* 1). ^7^Did not attend for endoscopy. (B) UVa and CCHMC study flow. ^1^For UVa: 1–18-y olds presenting for esophagogastroduodenoscopy for clinical diagnostic purposes at the University of Virginia University Hospital. For CCHMC: <12-y olds presenting for esophagogastroduodenoscopy for clinical diagnostic purposes at CCHMC without a comorbidity. ^2^Includes celiac disease under treatment (UVa *n =* 1), possible celiac disease (UVa *n =* 1), nonspecific duodenitis (UVa *n =* 1, CCHMC *n =* 1), gastritis (UVa *n =* 12, CCHMC *n =* 2), eosinophils in esophagus without conclusive diagnosis of EOE (UVa *n =* 3), *Helicobacter* (UVa *n =* 1), on treatment of possible eosinophilic esophagitis (UVa *n =* 1), biopsy contained gastric tissue only (UVa *n =* 1). BEECH, Biomarkers of Environmental Enteropathy in Children; BEED, Bangladesh Environmental Enteric Dysfunction; CCHMC, Cincinnati Children’s Hospital Medical Center; H+E Slide, hematoxylin and eosin stain slide; LTFU, lost to follow-up; PT INR, prothrombin time international normalized ratio; SEEM, Study of Environmental Enteropathy and Malnutrition; TTG IgA, tissue transglutaminase IgA; UVa, University of Virginia; WAZ, weight-for-age *z*-score.

The Consortium goal was to augment the contributions of the individual centers to answer high-level questions amenable to interrogation across the studies. We parsed these interrogations into 7 domains (Table 3) [[33], [34], [35], [36], [37], [38], [39]]. These are individually described and their data are presented in separate papers in this supplement. Previously published articles containing data from single-center studies that are related to papers in this supplemental issue are presented in Table 4 [[40], [41], [42], [43], [44], [45], [46], [47], [48], [49], [50], [51], [52], [53], [54], [55], [56], [57], [58], [59], [60], [61]]. Table 5 presents center-specific procedures and protocols, as participants progressed through recruitment, nutritional rehabilitation, evaluation, and, in a final subset, endoscopic biopsies. Each of the three centers used community-based enrollment to identify children meeting their anthropometry-based criteria to receive nutritional intervention. Bangladesh Environmental Enteric Dysfunction (BEED) enrolled Bangladeshi children on the basis of LAZ, falling into two groups: *1*) stunted (LAZ < −2) and *2*) “at risk of stunting” (LAZ ≥ −2 but < −1). These children received egg, milk, and micronutrient supplementation and if their LAZ did not improve compared with the LAZ category in which they started, they were eligible for endoscopy (assuming they did not have a history of chronic conditions, which would have made them ineligible for the nutritional intervention). Study of Environmental Enteropathy and Malnutrition (SEEM) enrolled Pakistani children on the basis of weight-for-length *z*-score (WLZ) < −2—these children received a ready-to-use-supplementary food. If their wasting status did not improve and no medical condition was identified, they were evaluated by a pediatric gastroenterologist to determine eligibility criteria for endoscopy. Four SEEM children had mildly elevated (1.01- to 2.07-fold greater than the upper limit of normal for the assay) serum concentrations of tissue transglutaminase (TTG) IgA on their initial screen tested within three days of the biopsy. However, because their TTG IgA concentrations were normal ∼2 y later while consuming a local traditional diet, which typically contains gluten, they were considered not to have celiac disease and were retained in the analyses as having EED at the time of the biopsy. Biomarkers of Environmental and Enteropathy in Children (BEECH) enrolled Zambian children on the basis of LAZ, weight-for-length *z*-score, or weight-for-age *z*-score of < −2. These children received high-energy protein supplementation, eggs, and micronutrient sachets. If their LAZ or WLZ was consistently <−2 after 3–4 mo of nutritional intervention, and their medical evaluation was noncontributory, they were eligible for endoscopy. Cincinnati Children’s Hospital Medical Center (CCHMC) and University of Virginia (UVa) enrolled children presenting for endoscopy and upper intestinal biopsy for clinical reasons up to ages 11 and 18 y, respectively. These children fell into 2 categories: *1*) newly diagnosed celiac disease and *2*) nondiagnostic group—those without medical diagnoses or diagnostic histology consistent with esophagogastrointestinal disease (including eosinophilic esophagitis, gastritis, and celiac and inflammatory bowel diseases); three children retained in the nondiagnostic group had incidental findings [esophagitis without eosinophilic infiltration (*n =* 2) and colonic polyp (*n =* 1)]. Figure 1A and B presents the recruitment flow and final enrollment by center and Table 6 displays demographic characteristics of the children who underwent endoscopy and were included in the final Consortium cohorts. Children enrolled at the three EED sites were of similar ages at the time of biopsies—the median (IQR) of ages were as follows: 1.6 (1.4–1.7) y for BEED, 1.7 (1.3–1.8) y for SEEM, 1.6 (1.3–1.8) y for BEECH. Because of the clinical indications for endoscopy at the United States centers, it is not surprising that these children were older [median (IQR) 6.8 (5.2–10.0) y].

**Table 3:** Environmental Enteric Dysfunction Biopsy Initiative Consortium domains

Domain	Objective(s)	Includes data from the following study centers	Paper in the Supplement Series
Histology	Refinement and validation of an EED histology scoring system	BEED, SEEM, BEECH, UVa, CCHMC	1. Histopathology underlying environmental enteric dysfunction in a cohort study of undernourished children in Bangladesh, Pakistan, and Zambia compared with United States children [33]
	Histologic features of EED compared to non-diagnostic and celiac disease comparison groups		
	Histologic differences between EED centers	BEED, SEEM, BEECH	
	Histologic differences between biopsies collected at different sites in the duodenum		
	Intra-individual differences in EED histology		
Immunohistochemistry (IHC)	Comparison of IHC quantitative measurements between EED to non-diagnostic and celiac disease comparison groups	BEED, SEEM, BEECH, UVa, CCHMC	2. Multiplexed immunohistochemical evaluation of small bowel inflammatory and epithelial parameters in environmental enteric dysfunction [36]
	Association of IHC markers to histology among children with EED	BEED, SEEM, BEECH	
Morphometry	Comparison of quantitative mucosal morphometric measurements between EED to non-diagnostic and celiac disease comparison groups	BEED, SEEM, BEECH, CCHMC	3. Duodenal quantitative mucosal morphometry in children with environmental enteric dysfunction: a cross-sectional multicountry analysis [37]
	Association of quantitative mucosal morphometric measurements to select histology and IHC markers among children with EED	BEED, SEEM, BEECH	
Transcriptomics	Small bowel tissue transcriptomic profiles in EED compared to non-diagnostic comparison group	BEED, SEEM, BEECH, UVa, CCHMC	4. Duodenal transcriptomics demonstrates signatures of tissue inflammation and immune cell infiltration in children with environmental enteric dysfunction across global centers [38]
	Small bowel tissue transcriptomic profile differences with severe vs less severe histology and specific histologic features among children with EED	BEED, SEEM, BEECH	
Anthropometry	Anthropometry association with histology	BEED, SEEM, BEECH, CCHMC, UVa	5. Anthropometry relationship with duodenal histological features of children with environmental enteric dysfunction: a multicenter cross-sectional study [39]
Biomarkers	Dual sugar permeability test and fecal biomarkers association with histology among children with EED	BEED, SEEM, BEECH	6. Biomarker relationships with small bowel histopathology among malnourished children with environmental enteric dysfunction in a multicountry cohort study [35]
Enteric Pathogens	Asymptomatic fecal enteric pathogen detection association with histology among children with EED	BEED, SEEM, BEECH	7. Enteric pathogens relationship with small bowel histological features of environmental enteric dysfunction in a multicountry cohort study [34]

Abbreviations: BEECH, Biomarkers of Environmental Enteropathy in Children; BEED, Bangladesh Environmental Enteric Dysfunction; CCHMC, Cincinnati Children’s Hospital Medical Center; EED, environmental enteric dysfunction; SEEM, Study of Environmental Enteropathy and Malnutrition; UVa, University of Virginia.

**TABLE 4:** Previously published articles containing data from single-center studies that are related to papers in this supplemental issue

Prior publication as of December 1, 2022	Topic and paper in this supplement						
	Introduction and background (this paper)	Histology [33]	Morphometry [37]	Transcriptomics [38]	Anthropometry [39]	Biomarkers [35]	Pathogens [34]
Ahmed et al. [40]						√	
Ahmed et al. [41]	√						
Amadi et al. [42]		√	√	√		√	√
Chandwe et al. [43]	√						
Chen et al. [44]		√		√	√	√	
Das et al. [45]					√	√	
Fahim et al. [46]					√	√	√
Gaffar et al. [47]					√		
Gaffar et al. [48]					√		
Gazi et al. [49]		√			√	√	
Gazi et al. [50]					√	√	
Haberman et al. [51]		√		√	√	√	
Hasan et al. [52]					√	√	
Hossain et al. [53]		√					
Hossain et al. [54]					√	√	
Iqbal et al. [55]	√						
Jamil et al. [56]		√			√	√	
Mahfuz et al. [57]					√		
Mahfuz et al. [58]	√						
Mulenga et al. [59]		√	√				
Zhao et al. [60]		√			√	√	
Zyambo et al. [61]		√	√	√		√

**TABLE 5:** Enrollment procedures by center

Center	Enrollment period Recruitment location	Recruitment strategy and eligibility criteria for nutritional intervention	Exclusion criteria for nutrition intervention	Nutritional intervention and nonresponse definition	Medical evaluation if failed nutritional intervention to exclude other causes of growth failure	Endoscopy timeframe
BEECH, Zambia	October 1, 2016–May 31, 2018 Urban slum in Misisi, Kuku, Chawama, and John Laing areas of Lusaka, Zambia	Door-to-door screening 1–18 mo-olds with WAZ < −1 invited for clinic-based extended anthropometry; eligible if WAZ, LAZ, or WLZ < −2	Caregiver unwilling for child to undergo HIV test and receive HIV care (if relevant), participating in another research study	Counseling on nutrition, water/sanitation/hygiene, and home care of child illness. Breastfeeding support and education. Starting at age 6 mo, high-energy protein supplement (corn–soya blend), 14 eggs, and 14 sachets of micronutrient powder provided every 2 wk. Children with complicated SAM received hospital management and those with uncomplicated SAM were managed with ready-to-use therapeutic feeds on an outpatient basis per national protocols Nonresponse: LAZ or WLZ consistently < −2 after 3–4 mo of nutritional supplementation	Examination by a pediatric gastroenterologist. Not eligible if chronic health condition that could cause growth faltering (e.g., cardiac disease), elevated tissue transglutaminase IgA suggestive of celiac disease, current history of diarrhea, currently on antibiotics, hemoglobin < 9 g/dL, leukocytosis, elevated PT INR	February 2, 2017–June 7, 2019
BEED, Bangladesh	July 17, 2016–May 31, 2019 Urban slum in Baunibadh and Mirpur areas of Dhaka, Bangladesh	Door-to-door screening 12–18 mo-olds with LAZ < −1	Severe acute malnutrition History of persistent diarrhea Known allergy to eggs or milk or milk intolerance	Directly observed on-site feeding of boiled egg and 150 mL whole milk at a study nutrition center 6 d/wk for 90 d. Also received antihelminthic treatment per national guideline, micronutrient sprinkles (1 sachet daily for 2 mo), and nutritional counseling for caregivers Nonresponse: For children with starting LAZ < −2, LAZ remains < −2. For children with starting LAZ < −1 but ≥ −2, LAZ remains < −1	Examination by a study physician. Ineligible if chronic health condition identified that could cause growth faltering (e.g., tuberculosis), elevated tissue transglutaminase IgA suggestive of celiac disease, hemoglobin < 8 g/dL.^1^ Before endoscopy, examination and review of preanesthesia laboratory results (clotting time, bleeding time, and PT INR) by a pediatric anesthetist. Ineligible if preanesthesia laboratory results were abnormal.^1^ If acute diarrhea, endoscopy deferred until resolved	November 2, 2016–August 26, 2019
SEEM, Pakistan	March 1 2016–November 2017 Matiari—a rural area 185 km from Karachi, Pakistan	Door-to-door screening Eligible for monitoring if 3–6 mo old and WLZ < −2. Nutritional counseling provided. If WLZ still < −2 at 9 mo, eligible for nutrition intervention	WLZ > 0 and LAZ not < −1 on 2 consecutive visits	Nutritional counseling for caregivers. Starting at age 9 mo, AchaMuM—a ready-to-use supplementary food—1 sachet daily for 2 mo for WLZ < −2 but ≥ −3. For WLZ < −3, weight-based supply was provided Nonresponse: no improvement in length and weight of child compared with preceding weight and length	Examination by 2 pediatricians after the completion of nutrition intervention. Ineligible if >24 mo of age or if chronic health condition identified that could cause growth faltering (e.g., neurologic or cardiac disorders, tuberculosis). Four children had elevated serum concentrations of tissue transglutaminase IgA. Repeat concentrations were normal while consuming a local traditional diet, which typically contains gluten; data from these children were retained in the SEEM cohort and Consortium analyses. Ineligible if hemoglobin < 8 g/dL, elevated PT INR,^2^ or thrombocytopenia	January 11, 2017–October 10, 2018
CCHMC, United States	March 24, 2017–March 5, 2019 Cincinnati Children’s Hospital Medical Center Cincinnati, Ohio	Patients <12 y of age presenting for endoscopic intestinal biopsy for diagnostic purposes.^3^ Included 2 cohorts: *1*) newly diagnosed celiac disease and *2*) nondiagnostic group who had no medical diagnoses or diagnostic histology consistent with esophagogastrointestinal disease^4^	—	—	—	March 24, 2017–March 5, 2019
UVa, United States	June 7, 2017–September 13, 2019 University of Virginia University Hospital Charlottesville, Virginia	Patients aged 1–18 y presenting for endoscopic intestinal biopsy for diagnostic purposes.^3^ Included 2 cohorts: *1*) newly diagnosed celiac disease and *2*) nondiagnostic group who had no medical diagnoses or diagnostic histology consistent with esophagogastrointestinal disease^5^	—	—	—	June 7, 2017–September 13, 2019

Abbreviations: LAZ, length-for-age *z*-score; PT INR, prothrombin time international normalized ratio; SAM, severe acute malnutrition; WAZ, weight-for-age *z*-score; WLZ, weight-for-length *z*-score.

1 Per protocol, although no child had a hemoglobin <8 g/dL or an abnormal clotting time, bleeding time, or PT INR.

2 Per protocol, although no child had a hemoglobin <8 g/dL or an abnormal PT INR.

3 Nutritional intervention does not apply to the United States cohorts.

4 Children diagnosed with definitive or suspected eosinophilic esophagitis, gastritis, and inflammatory bowel diseases were excluded; 2 children had incidental findings of esophagitis without eosinophilic infiltration and were retained in the nondiagnostic cohort.

5 Children diagnosed with definitive or suspected eosinophilic esophagitis, gastritis, and inflammatory bowel diseases were excluded; 1 child had an incidental finding of a colonic polyp and was retained in the nondiagnostic cohort.

**TABLE 6:** Demographic characteristics of participants by center and disease status

	BEED *N =* 120	SEEM *N =* 63	BEECH *N =* 108	EED studies combined *N =* 291	UVa Celiac *N =* 2	CCHMC Celiac *N =* 20	United States Celiac combined *N =* 22	UVa Nondiagnostic *N =* 16	CCHMC Nondiagnostic *N =* 28	United States Nondiagnostic combined *N =* 44
Age^1^ (y) median (IQR)	1.6 (1.4, 1.7)	1.7 (1.3, 1.8)	1.6 (1.3, 1.8)	1.6 (1.4, 1.8)	6.3 (4.5, 8.1)	7.1 (5.2, 10.1)	7.1 (5.2, 10.0)	12.1 (7.6, 15.6)	5.5 (4.2, 6.7)	6.7 (5.2, 9.5)
Female sex (%)	58.3%	30.2%	50.9%	49.5%	50%	55%	54.5%	50%	42.9%	45.5%
HAZ/LAZ^2^ median (IQR)	−2.1 (−2.8, −1.5)	−3.2 (−3.7, −2.4)	−3.3 (−3.9, −2.8)	−2.8 (−3.5, −2.1)	0.9 (0.1, 1.7)	0.1 (−0.8, 0.4)	0.1 (−0.7, 0.4)	0.7 (−0.5, 1.6)	0.0 (−1.0, 1.2)	0.2 (−0.9, 1.3)
WAZ^2^ median (IQR)	−1.7 (−2.3, −1.2)	−3.1 (−3.6, −2.6)	−2.2 (−2.7, −1.8)	−2.2 (−2.8, −1.6)	1.1 (0.9, 1.3)	0.5 (0.4, 0.8)	0.5 (0.5, 0.9)	0.3 (0.2, 0.6)	0.0 (0.0, 0.5)	0.1 (0.0, 0.5)
WHZ/WLZ^2^^,^^3^ median (IQR)	−1.0 (−1.4, −0.4)	−2.2 (−2.8, −1.8)	−0.8 (−1.3, −0.2)	−1.1 (−1.8, −0.5)	−0.6 (−0.6, −0.6)	−0.2 (−0.7, 0.4)	−0.6 (−0.6, 0.2)	—	−0.6 (−1.0, 0.0)	−0.6 (−1.0, 0.0)
BMI %ile^2^^,^^4^ median (IQR)	—	—	15.0 (14.7, 15.5)	15.0 (14.7, 15.5)	19.0 (17.0, 20.9)	15.4 (14.7, 16.4)	15.4 (14.8, 16.5)	18.4 (16.2, 23.4)	15.4 (14.2, 16.5)	15.6 (14.8, 18.6)

Abbreviations: BEECH, Biomarkers of Environmental Enteropathy in Children; BEED, Bangladesh Environmental Enteric Dysfunction; CCHMC, Cincinnati Children’s Hospital Medical Center; EED, environmental enteric dysfunction; HAZ, height-for-age *z*-score; LAZ, length-for-age *z*-score; SEEM, Study of Environmental Enteropathy and Malnutrition; UVa, University of Virginia; WAZ, weight-for-age *z*-score; WHZ, weight-for-height *z*-score; WLZ, weight-for-length *z*-score.

1 At the time of biopsy.

2 Measurements closest to the time of biopsy.

3 WHZ/WLZ can only be calculated for children aged <5 y. All EED cohorts included children aged <5 y. CCHMC Celiac cohort included 4, UVa Celiac included 1, CCHMC Nondiagnostic included 10, and UVa Nondiagnostic included 0.

4 BMI is used as a measure of thinness (or overweight status) among children aged ≥2 y. All United States cohorts included children aged ≥2 y. BEECH cohort included 11 children aged ≥2 y at the time of biopsy.

## Duodenal Endoscopy Biopsy

Endoscopy of the upper gastrointestinal tract was performed per standards recommended by the American Society for Gastrointestinal Endoscopy, and the European Society for Paediatric Gastroenterology, Hepatology and Nutrition [62,63].

Each participant underwent a pre-endoscopy risk assessment and anesthetic evaluation. Endoscopy did not proceed in those children deemed unfit for the procedure as a result of significant medical contraindications (for example, severe anemia, coryza, tachypnea, severe diarrhea, coagulopathy, or thrombocytopenia). After the procedure, participants were closely observed until they had regained full consciousness. No serious complications were encountered as a result of endoscopy in any of the participants in this study.

Further details on biopsy locations within the duodenum are described in Kelly et al. [33] in this supplement. All biopsies were paraffin-embedded, sectioned, and stained with hematoxylin and eosin (H&E). H&E slides were scanned on site or transported to Washington University in St. Louis for scanning at 40×. All whole-slide images were uploaded to the Washington University Digital Pathology Exchange telepathology platform [32].

## Ethical Approvals

Signed informed consent from parent/legal guardian was obtained at enrollment into the study for the growth monitoring and nutritional intervention portion and separately for endoscopy and biopsy (BEED, SEEM, and BEECH). Informed consent from parent/legal guardian of the children presenting at CCHMC and UVa was obtained for study participation including the analysis of clinical data and histology obtained for clinical purposes as well as obtaining additional intestinal biopsies for research purposes. Ethical approval was obtained from AKU Ethics Review Committee (ERC) (3836-Ped-ERC-15), icddr,b ERC (PR-16007), University of Zambia Biomedical Research Ethics Committee (006-02-16) and National Health Research Authority (MH/101/23/10/1), UVa Institutional Review Board (19466), and CCHMC Institutional Review Board (2016-0387). Exemption was received from the University of Washington Institutional Review Board (IRB) (STUDY00013442) and the Washington University IRB (201801207).

## Additional Specimens Sought

In addition to duodenal biopsies, the BEED, SEEM, and BEECH studies collected stool for fecal biomarkers and enteric pathogen identification and the BEED and SEEM studies collected urines as part of dual sugar permeability testing. Collection timing and other relevant details are presented in the respective papers in this series [34,35].

## Conclusion

It is remarkable that much of our prior understanding of the mucosal pathophysiology of pediatric EED relies on biopsies obtained decades ago from small cohorts of children with diarrhea, marasmus, or kwashiorkor [14,64,65]. Clearly, there has been a profound contrast between our knowledge of the organ pathology in EED, and our understanding of the pathophysiology of the process. Our work attempts to fill this void. In this series of papers, the EEDBI Consortium applies standard clinical H&E histology, traditional quantitative mucosal morphometry, advanced tissue transcriptomics and IHC, and assessment of less invasively obtained specimens to provide an unprecedented analysis of a disorder that has profound implications for child health across three EED-endemic geographically distinct locations. In the first paper, we present a novel semiquantitative histologic scoring system that can be applied in future studies and describe its application within the EEDBI Consortium data to highlight histologic characteristics of the disorder, tease out differences between EED centers, and compare features to another enteropathy—celiac disease [33]. The second paper presents IHC analyses that begin to shed light on functional characteristics of EED [36]. The third paper in the series interrogates villus crypt structure assessed by mucosal morphometry and relates this quantification to select histologic and immunohistochemical features [38]. The fourth paper integrates mechanistic insights by applying transcriptomics to the biopsy specimens of the EED and comparison groups [38]. Transcript associations with histologic severity and specific histologic parameters among the children with EED are also assessed. The fifth paper explores relationships between anthropometry and histology [39]. The sixth paper examines associations between histology and biomarkers—urinary dual sugar permeability measures and fecal markers of intestinal inflammation [35]. The seventh paper explores the role that asymptomatic pathogen carriage may play in inciting a histologic response [34].

## Author contributions

The authors’ responsibilities were as follows – TA, SAA, BA, PK, MM, CM, SRM, WAP: designed the research; SA, TA, SAA, BA, PK, MM, CM, SRM, WAP: conducted the research; DMD, SL, JPN, PBS, PIT: data management; DMD, JPN, PBS, PIT: data interpretation; SL: telepathology management; JPN: edited the paper; DMD, SL, PBS, PIT: wrote the paper; DMD: had primary responsibility for final content; and all authors: read and approved the final manuscript.

### Conflict of interest

The authors report no conflicts of interest.

## Funding

The EEDBI Consortium was funded by the following grants: Bill and Melinda Gates Foundation OPP1152812, OPP1066118, OPP1136759, OPP1138727, and OPP1144149, and Advanced Imaging and Tissue Analysis Core of the Washington University Digestive Diseases Research Core Center P30DK052574.

## Data availability

Data described in the manuscript, code book, and analytic code will be made available upon request to the corresponding author pending application and approval.

## Figures and Tables

**Table:** 

Surname	Name	Full affiliation	E-mail
Ahmed	Kumail	Department of Paediatrics and Child Health, Aga Khan University, Karachi, Pakistan	kumail.ahmed@aku.edu
Alam	Md Ashraful	Nutrition Research Division, International Centre for Diarrhoeal Disease Research, Bangladesh, Dhaka, Bangladesh	mashraful@icddrb.org
Barnes	Barrett H	Department of Pediatrics, University of Virginia Children's Hospital, University of Virginia, Charlottesville, VA, United States	bhb6u@virginia.edu
Begum	SM Khodeza Nahar	Department of Pathology, Bangladesh Specialized Hospital, Dhaka, Bangladesh	khodeza33@hotmail.com
Borowitz	Stephen M	Department of Pediatrics, University of Virginia, Charlottesville, VA, United States	smb4v@virginia.edu
Chandwe	Kanta	Tropical Gastroenterology & Nutrition Group, University of Zambia, Lusaka, Zambia	kantachandwe@gmail.com
Chipunza	Miyoba	Tropical Gastroenterology & Nutrition Group, University of Zambia, Lusaka, Zambia	miyobachipunza@gmail.com
Das	Subhasish	Nutrition Research Division, International Centre for Diarrhoeal Disease Research, Bangladesh, Dhaka, Bangladesh	subhasish.das@icddrb.org
Denson	Lee A	Division of Pediatric Gastroenterology, Hepatology, and Nutrition, Cincinnati Children's Hospital Medical Center, Cincinnati, OH, United States	Lee.Denson@cchmc.org
Donowitz	Jeffrey R	Division of Pediatric Infectious Diseases, Children's Hospital of Richmond at Virginia Commonwealth University, Richmond, VA, United States	jeffrey.donowitz@vcuhealth.org
Fahim	Shah Mohammad	Nutrition Research Division, International Centre for Diarrhoeal Disease Research, Bangladesh, Dhaka, Bangladesh	mohammad.fahim@icddrb.org
Gazi	Md Amran	Nutrition Research Division, International Centre for Diarrhoeal Disease Research, Bangladesh, Dhaka, Bangladesh	amran.gazi@icddrb.org
Gilchrist	Carol A	Department of Medicine, University of Virginia, Charlottesville, VA, United States	cg2p@virginia.edu
Haque	Rashidul	Infectious Diseases Division, International Centre for Diarrhoeal Disease Research, Bangladesh, Dhaka, Bangladesh	rhaque@icddrb.org
Hasan	Md Mehedi	Nutrition Research Division, International Centre for Diarrhoeal Disease Research, Bangladesh, Dhaka, Bangladesh	md.hasan@icddrb.org
Hossain	Md Shabab	Nutrition Research Division, International Centre for Diarrhoeal Disease Research, Bangladesh, Dhaka, Bangladesh	dr.shabab@icddrb.org
Hotwani	Aneeta	Department of Paediatrics and Child Health, Aga Khan University, Karachi, Pakistan	aneeta.hotwani@aku.edu
Iqbal	Junaid	Department of Paediatrics and Child Health, Aga Khan University, Karachi, Pakistan	junaid.iqbal@aku.edu
Iqbal	Najeeha Talat	Department of Paediatrics and Child Health, Aga Khan University, Karachi, Pakistan	najeeha.iqbal@aku.edu
Jakhro	Sadaf	Department of Paediatrics and Child Health, Aga Khan University, Karachi, Pakistan	sadaf.jakhro@aku.edu
Kabir	Furqan	Department of Paediatrics and Child Health, Aga Khan University, Karachi, Pakistan	furqan.kabir@aku.edu
Mann	Barbara J	Department of Medicine, University of Virginia, Charlottesville, VA, United States	bjm2r@virginia.edu
Mazumder	Ramendra Nath	Nutrition Research Division, International Centre for Diarrhoeal Disease Research, Bangladesh, Dhaka, Bangladesh	ramen@icddrb.org
Memon	Waheeda	Department of Paediatrics and Child Health, Aga Khan University, Karachi, Pakistan	waheeda.memon@aku.edu
Middleton	Jeremy P	Department of Pediatrics, University of Virginia, Charlottesville, VA, United States	jpm8k@virginia.edu
Nayak	Uma	Department of Public Health Sciences, University of Virginia, Charlottesville, VA, United States	un8x@virginia.edu
Oliphant	Sandra	Department of Pediatrics, University of Virginia, Charlottesville, VA, United States	bjm2r@virginia.edu
Qureshi	Abdul Khalique	Department of Paediatrics and Child Health, Aga Khan University, Karachi, Pakistan	abdul.qureshi@aku.edu
Rahman	Masudur	Department of Gastroenterology, Sheikh Russel National Gastroliver Institute and Hospital, Dhaka, Bangladesh	drmasud47@yahoo.com
Rahman	Najeeb	Department of Paediatrics and Child Health, Aga Khan University, Karachi, Pakistan	najeeb.rahman@aku.edu
Ramakrishnan	Girija	Department of Medicine, University of Virginia, Charlottesville, VA, United States	gr6q@virginia.edu
Sadiq	Kamran	Department of Paediatrics and Child Health, Aga Khan University, Karachi, Pakistan	kamran.sadiq@aku.edu
Sarker	Shafiqul Alam	Nutrition Research Division, International Centre for Diarrhoeal Disease Research, Bangladesh, Dhaka, Bangladesh	sasarker@icddrb.org
Umrani	Fayaz	Department of Paediatrics and Child Health, Aga Khan University, Karachi, Pakistan	fayaz.umrani@aku.edu
